# Biological Convergence of Cancer Signatures

**DOI:** 10.1371/journal.pone.0004544

**Published:** 2009-02-20

**Authors:** Xavier Solé, Núria Bonifaci, Núria López-Bigas, Antoni Berenguer, Pilar Hernández, Oscar Reina, Christopher A. Maxwell, Helena Aguilar, Ander Urruticoechea, Silvia de Sanjosé, Francesc Comellas, Gabriel Capellá, Víctor Moreno, Miguel Angel Pujana

**Affiliations:** 1 Bioinformatics and Biostatistics Unit, Catalan Institute of Oncology, IDIBELL, L'Hospitalet, Barcelona, Spain; 2 Translational Research Laboratory, Catalan Institute of Oncology, IDIBELL, L'Hospitalet, Barcelona, Spain; 3 Research Unit on Biomedical Informatics of IMIM/UPF, Barcelona Biomedical Research Park, Barcelona, Spain; 4 Unit of Infections and Cancer, CIBERESP, Epidemiology Research of Cancer Program, Catalan Institute of Oncology, IDIBELL, L'Hospitalet, Barcelona, Spain; 5 Department of Applied Mathematics IV, Technical University of Catalonia, Castelldefels, Barcelona, Spain; IBM Thomas J. Watson Research Center, United States of America

## Abstract

Gene expression profiling has identified cancer prognostic and predictive signatures with superior performance to conventional histopathological or clinical parameters. Consequently, signatures are being incorporated into clinical practice and will soon influence everyday decisions in oncology. However, the slight overlap in the gene identity between signatures for the same cancer type or condition raises questions about their biological and clinical implications. To clarify these issues, better understanding of the molecular properties and possible interactions underlying apparently dissimilar signatures is needed. Here, we evaluated whether the signatures of 24 independent studies are related at the genome, transcriptome or proteome levels. Significant associations were consistently observed across these molecular layers, which suggest the existence of a common cancer cell phenotype. Convergence on cell proliferation and death supports the pivotal involvement of these processes in prognosis, metastasis and treatment response. In addition, functional and molecular associations were identified with the immune response in different cancer types and conditions that complement the contribution of cell proliferation and death. Examination of additional, independent, cancer datasets corroborated our observations. This study proposes a comprehensive strategy for interpreting cancer signatures that reveals common design principles and systems-level properties.

## Introduction

Recent years have seen the description of a large number of gene expression profiles or signatures with clinical value for the accurate prognostic or predictive characterization of cancer patients or tumors. Breast cancer is probably the paradigm of such studies, with at least three different signatures currently being tested in clinical trials and commercially available for routine clinical practice in oncology [1], [2]. However, the lack of overlap in the selected genes has raised fundamental questions about their biological and clinical implications [3], [4]. This situation is not unique to breast cancer prognosis, and the description of new expression profiles suggests that it is common to other cancer types or conditions―i.e. metastases and treatments [5]. Reasons to this paradox may be methodological disparities [6] and statistical constraints created by the large number of genes examined with respect to the relatively small number of samples profiled [7]–[9]. Importantly, a recent study by Perou and colleagues [10] established the common prognostic value of some breast cancer signatures, despite the lack of overlap in gene identities. This observation confirmed the clinical relevance of the signatures and suggested that they may efficiently capture a common tumor cell phenotype(s) [11]. This putative common phenotype for breast cancer and for other neoplasias must be defined if we are to better understand the significance of signatures.

Some of the early descriptions of signatures noted the presence of specific biological processes over-represented in the corresponding gene lists. Among these processes, individual genes involved in the cell cycle and apoptosis were highlighted (e.g. [12], [13]). More recent evidence points to specific genes that are globally associated with breast cancer prognosis and related to cell proliferation among other processes or pathways [14]–[21]. However, it is still unclear how this evidence characterizes different molecular levels and how the levels integrate into a systems-level model containing gene and/or protein interactions for breast cancer and for human cancer in general. Here, we used an integrative approach to determine the existence of a putative common tumor cell phenotype(s) associated with different cancer types and conditions. The study identified common molecular properties and network interactions associated with cell proliferation and death, and revealed associations with the immune response. Our results highlight the importance of studying signatures from a systems-level perpective.

## Results

### Genomic properties: E2Fs and the estrogen receptor (ER)

**Figure 1:**
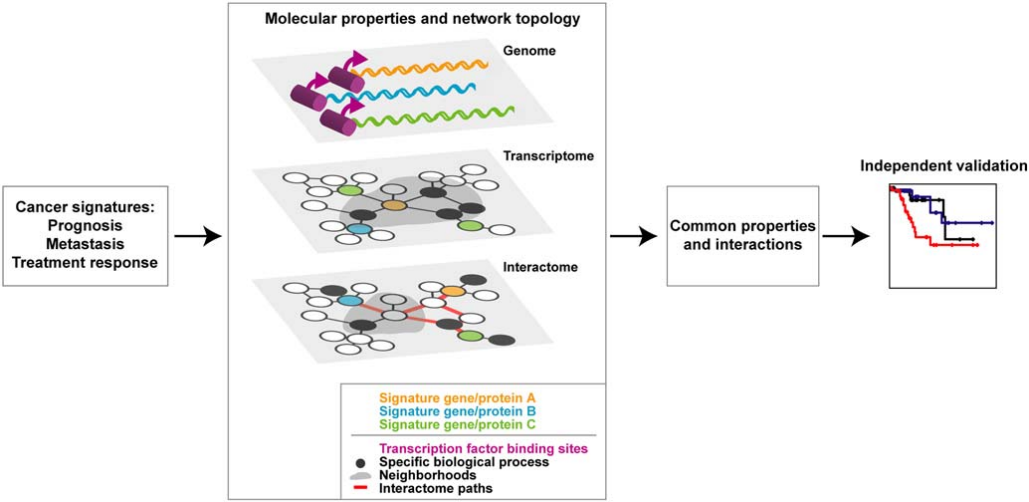
Integrative analysis of cancer signatures. Strategy for defining the common properties and interactions between signatures at the genome, transcriptome and proteome levels, and validation in independent datasets.

To identify common properties among cancer signatures we compiled the literature gene lists from 24 studies (Table S1). These represent 19 prognostic signatures, two signatures focused mainly on metastasis, and seven predictive treatment response signatures. All signatures used corresponded to validated sets of genes at the same level. We first examined the molecular properties or network topology characteristics of genes and/or proteins in these signatures at the genome, transcriptome and proteome levels. Next, the identified properties and network associations were corroborated in independent expression datasets of different cancer types and conditions (Fig. 1).

**Figure 2:**
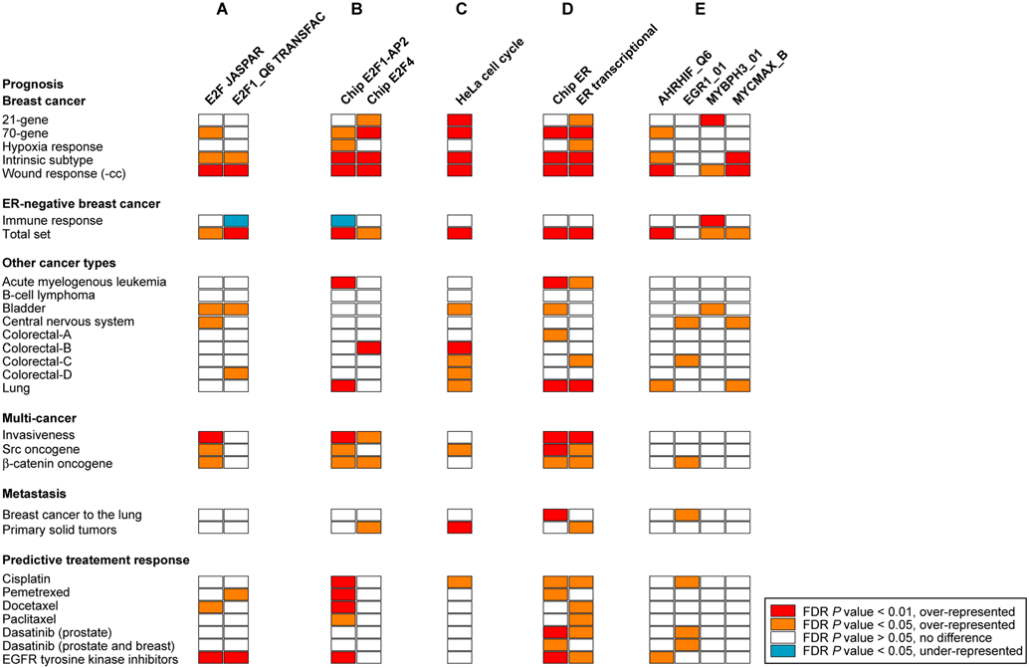
Genomic and transcriptomic properties of cancer signatures associated with the potential for cell proliferation and repressed cell death. *A*, representation of E2F motifs based on JASPAR and TRANSFAC matrices and the Poisson distribution, with *P* values adjusted using the FDR approach for analyses-columns. Values are shown as detailed in the inset: red/orange indicates significant over-representation and blue indicates significant under-representation. The E2F1_Q6 motif represents the putative action of E2F1 and MYC. *B*, representation of E2F1-AP2 and E2F4 binding sites from chromatin immunoprecipitation (chip) assays using the same statistical methodology as described above. The E2F4 data correspond to the joint analysis of cell cycle phases [23]. *C*, representation of genes with periodic expression through the cell cycle. *D*, representation of ER transcriptional regulation from chromatin immunoprecipitation assays or transcriptional changes in MCF7 cells. *E*, representation of additional promoter motifs using TRANSFAC matrices. The wound response signature without cell cycle-associated genes is indicated by the suffix “(-cc)”, and the “total set” signature of ER-negative breast cancer contains the immune response plus other biological processes such as the cell cycle. The dasatinib predictive signature is divided into two sets for the effect in prostate and breast cancer respectively. The colorectal prognostic signatures are as defined in Table S1.

Properties at the genome level were evaluated by probing the relative enrichment in predicted transcription factor binding site motifs at the promoters of signature genes (see Methods). In these analyses the top-ranked motifs across several signatures were from the E2F family. Significant over-representation of E2F motifs was identified in ∼45% (13/28) of the signatures tested, including prognostic (bladder, breast and central nervous system (CNS) cancers, and three multi-cancer signatures) and predictive signatures (docetaxel in breast tumors, EGFR tyrosine kinase inhibitors (TKIs) in lung tumors and pemetrexed in advanced solid tumors) (false discovery rate (FDR)-adjusted *P* values<0.05) (Fig. 2*A*). In contrast, only one signature (the immune response prognostic signature in estrogen receptor (ER)-negative breast cancer [22]) showed under-representation of E2F motifs. This observation will be discussed in the following sections.

To evaluate motif predictions in the promoter sequences of signature genes, we examined experimental data from chromatin immunoprecipitation assays of transcription factors [23], [24]. This analysis corroborated the major role of E2F transcriptional programs. Approximately 65% of signatures showed significant over-representation of E2F1-AP2 and/or E2F4 binding sites (Fig. 2*B*). The strongest over-representations were detected in prognostic―particularly breast cancer―and predictive treatment response signatures for E2F1-AP2 sites. Nevertheless, specificities were also suggested for the immune response, which showed under-representation of E2F1-AP2, and for predictive signatures that did not show differential representation of E2F4 in any case.

The E2Fs are key regulators of cell proliferation and death [25], [26], and common deregulation of E2F-mediated transcriptional programs is a hallmark of cancer transcriptomes [27]. The link with the potential for cell proliferation was further evaluated by examining transcripts with periodic expression through the cell cycle [28], which indicates a direct or indirect role in phase(s) of cell division, and by analyzing ER functional genomic data [29]. Significant over-representation of periodically expressed genes was observed in ∼45% of the signatures, most of which were prognostic signatures for different cancer types (Fig. 2*C*). Detailed examination of cell cycle phases showed specific over-representation of genes with an expression peak at G2 and G2/M, which is in agreement with their role in cell division (data not shown). In addition, consistent with the link between cell proliferation and the ER signaling pathway [30], significant over-representation of ER binding sites and/or ER-mediated transcriptional regulation was identified in most of the signatures (∼90%), irrespective of their type or condition (Fig. 2*D*). This high overlap with ER regulation probably reflects an strong association with cell proliferation beyond cancer hormone-dependencies.

Overall, all except two of the signatures examined here showed significant over-representation of one or more of the molecular evidences associated with the regulation of cell proliferation and death. The exceptions were the immune response signature, which may reflect the involvement of different biological processes, and the B-cell lymphoma prognosis signature, which may be explained by the statistical power needed to detect differences in the smallest gene set examined (*n* = 19). Similarities for these signatures at additional molecular levels will be presented in the following sections.

### Additional programs of cell proliferation, death and metastasis

In an examination for additional mechanisms of transcriptional regulation of signatures, motifs of AHR, EGR1, MYB and MYC were found to be over-represented in a second term. These over-representations were not as widespread as for E2Fs or ER, which suggests that they play only a minor role, but different cancer types and conditions were included: an EGR1 motif was found to be over-represented in CNS and colorectal cancers and the β-catenin multi-cancer prognostic signatures, the breast cancer lung metastasis signature and the predictive signatures of cisplatin and dasatinib (FDR-adjusted *P* values<0.05) (Fig. 2*E*). In agreement with these observations, we found the lung metastasis signature to contain 22% (12/54) of the genes predicted elsewhere to be EGR1 transcriptional targets [31]–[34] and the wound response was previously shown to be coordinated with *MYC* amplification [35]. In addition, over-representation of an AHR motif is consistent with its association with ER to regulate cell proliferation [36].

**Figure 3:**
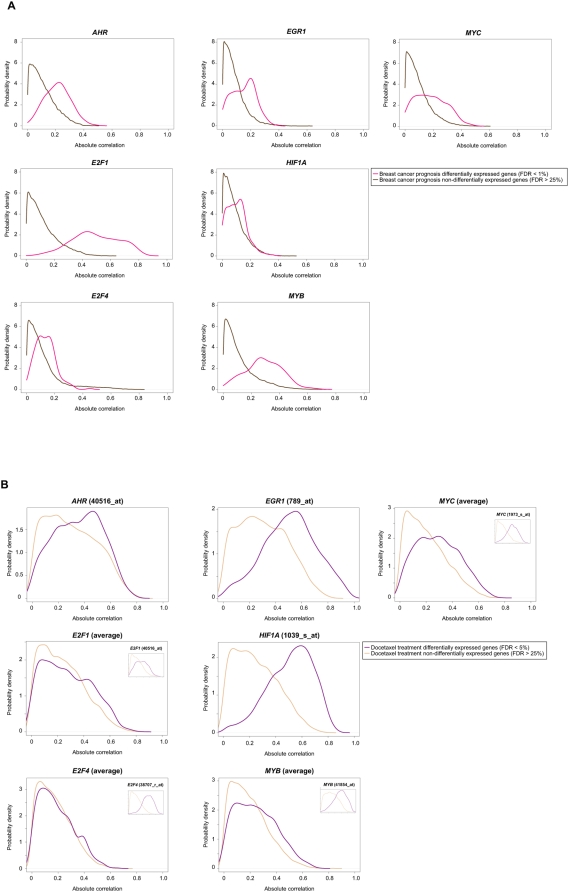
Expression correlations with defined transcription factors. *A*, expression correlations between seven transcription factors―gene names shown at the top of each graph―and genes differentially expressed for breast cancer prognosis measured by metastasis events up to 5 years (pink curves) relative to non-differentially expressed genes in this condition (brown curves). The graphs show absolute PCC values. *B*, same analysis for differentially expressed genes after docetaxel treatment of breast cancer patients relative to non-differentially expressed genes in this condition. Results for *E2F1*, *E2F4*, *MYB* and *MYC* are for average values of all microarray probes representing each factor, whereas the insets show the results for individual probes with significant differences.

Next, the significance of motif representations was evaluated by analyzing gene expression correlations in representative cancer datasets. Thus, we computed correlations using the Pearson correlation coefficient (PCC) between the seven transcription factors presented above and genes associated with breast cancer prognosis [12] or with the response to docetaxel treatment in breast cancer [37], and compared them with genes non-differentially expressed in these conditions. Higher absolute PCCs between transcription factors and genes associated with prognosis or treatment response were identified in all cases for genes and/or microarray probes (Mann-Whitney (MW) test *P* values<0.001) (Fig. 3). The prognosis dataset contained a single representative microarray probe for each transcription factor, therefore all of them showed significant differences (Fig. 3*A*). The treatment response dataset contained several probes for some factors, which were evaluated individually to identify technical or biological differences. In this dataset, *AHR*, *EGR1* and *HIF1A* were each represented by a single probe and all of them showed significantly higher correlations with response (Fig. 3*B*). *E2F1*, *E2F4*, *MYC* and *MYB* had more than one probe each, with discordant results in some cases but with average PCCs significantly associated with response in three of them (Fig. 3*B*), whereas *E2F4* remained unclassifiable as two probes were significantly correlated and two were not (data not shown).

To further evaluate these observations, we computed correlations between the seven transcription factors and 5,000 randomly selected sets equivalent to the size of the differentially expressed gene sets above. Higher PCCs were observed for most transcription factors in both cancer conditions, with the exception of *E2F4* in prognosis and treatment response (*P* values obtained using the empirical distribution of random PCCs (empirical *P* values) were of 0.16 and 0.11, respectively). Overall, the identification of significant correlations with at least six of the seven factors tested supports the motif predictions and suggests the existence of common transcriptional programs that converge on cell proliferation and death, as well as metastasis as revealed by EGR1 [33].

Analysis of motifs and expression correlations also revealed an association between the apparently dissimilar immune response set and different prognosis signatures. Although it under-represented E2F1 motifs, the immune response shared over-representation of a MYB motif with the 21-gene, wound response breast cancer, and bladder cancer prognostic signatures (Fig. 2*E*). Over-representation of this factor in the immune response is consistent with its role in hematopoiesis [38], and its over-representation in other signatures is consistent with the emerging involvement of the immune response in the prognosis of different cancer types [39]. The high correlations observed in Fig. 3*A* between *MYB* and genes globally associated with breast cancer prognosis (i.e. not limited by the ER status) support this hypothesis. Associations of this signature at other molecular levels will be presented in the following sections.

### Transcriptomic correlations between signatures

**Figure 4:**
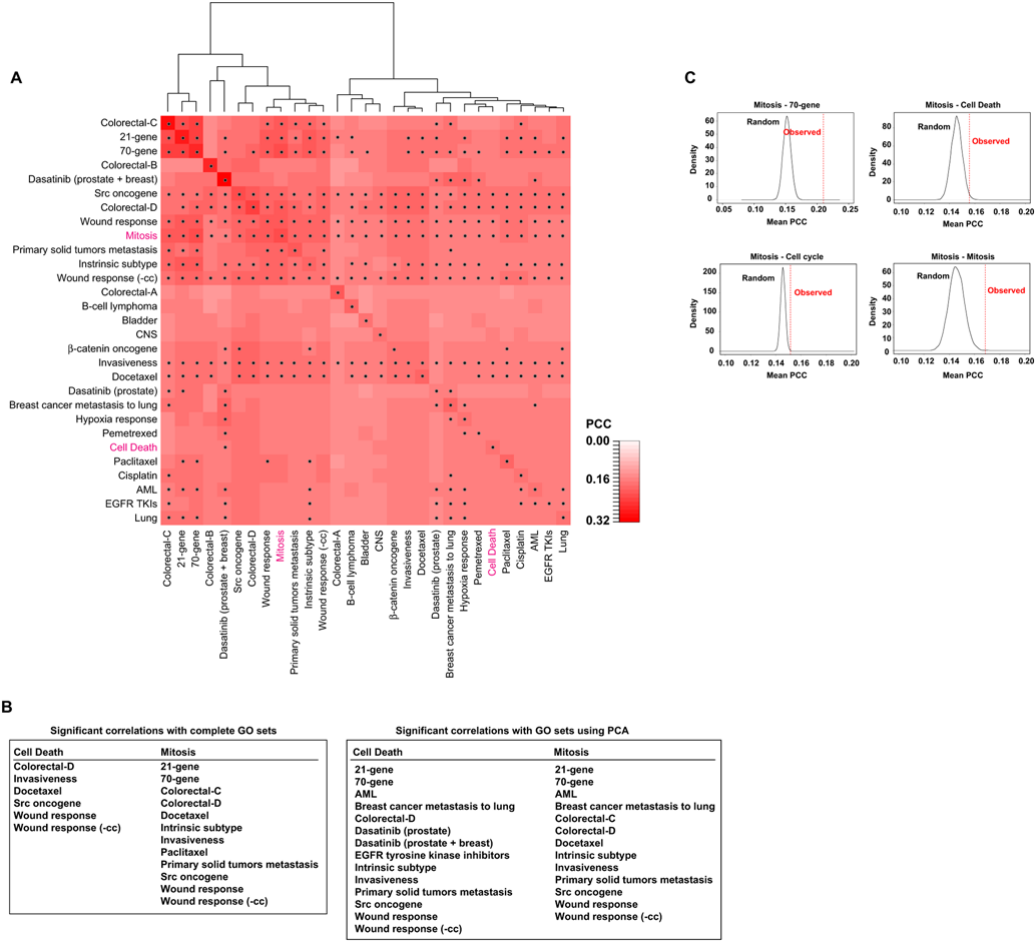
Transcriptomic correlations between signatures and with defined biological processes. *A*, heat map of average PCCs between cancer signatures in a breast cancer gene expression dataset [40]. Significant co-expression (empirical *P* values<0.05) is indicated by dots. Note that the matrix is not symmetrical because the results were dependent on the size of each gene set; therefore, the larger gene sets (e.g. wound response or invasiveness) showed significant co-expression with many other signatures, perhaps partly due to the fact that they had greater statistical power with which to detect them. Each dot corresponds to the comparison between a signature on the left (simulated set) and a signature at the bottom. The Cell Death and Mitosis sets are highlighted in pink. *B*, left panel, list of signatures that showed significant correlation with the Cell Death or Mitosis complete GO sets. Right panel, list of signatures that showed significant correlation with the Cell Death or Mitosis sets, but only using their principal components. *C*, observed (discontinuous red line) versus expected (black curve for 10,000 randomly selected sets) average PCCs between the Mitosis set and the 70-gene set, the Cell Death set, or genes with periodic expression through the cell cycle.

Given the identification of common transcriptional programs, global expression correlations between signatures should be higher than expected by chance. Using a breast cancer dataset [40] and the average PCC across all microarray probe pairs between any two signatures, significant co-expression was identified in approximately half of the analyses when compared to 10,000 equivalent, randomly selected gene sets (empirical *P* values<0.05) (Fig. 4*A*). These results support the existence of functional and molecular associations between many apparently dissimilar signatures, despite the fact that the dataset used had evident technical and biological specificities. Furthermore, the immune response signature showed significant co-expression with 15 of the signatures studied (data not shown), which also supports convergence on this process.

To further test the link to cell proliferation and death at the transcriptomic level, and excluding *a priori* information on expression levels or profiles that could bias the analysis, we examined correlations with gene sets selected using only the criteria for the Gene Ontology (GO) terms Cell Death and Mitosis. These sets were exclusively defined by selecting Entrez genes annotated with those terms, and then used in comparisons in the same way as any other signature. Using 10,000 equivalent random sets, absolute correlations between these GO sets and the signatures were found to be significantly higher in ≥12 comparisons (Fig. 4*B*, *left*). The Cell Death set was significantly correlated with five signatures and the Mitosis set was significantly correlated with 11 signatures of different cancer types or conditions. Importantly, differences in the GO sets relative to random were of the same magnitude as comparisons between signatures (Fig. 4*C*).

This analysis suggested that measuring the expression levels of genes known to participate in specific biological processes is likely to be of prognostic or predictive value in different situations. However, the analysis was constrained by the possible presence of non-informative expression or sub-sets of genes with different behavior within the GO sets. Thus, reducing the dimensionality of Cell Death and Mitosis sets using a principal component analysis that captured ∼80% of the variance raised the number of significant correlations to 12 and 14 sets, respectively (Fig. 4*B*, *right*); these numbers corresponded to a total of ∼60% of the signatures examined, irrespective of their type or condition.

### Interactome network associations

Functional relationships between proteins can be identified as direct interactions, complex memberships or relatively close connections in the network of protein-protein interactions or interactome network. Given the evidence at the genomic and transcriptomic levels presented above, we hypothesized that proteins encoded by apparently dissimilar signatures will be more closely located in the interactome network than expected by chance. For this analysis we used a dataset consisting mainly of experimentally identified protein-protein interactions, excluding homodimers and orthology-based predictions, and calculated the shortest path between any two nodes or proteins in the giant network component (i.e., the component containing the largest number of connected proteins) [41].

**Figure 5:**
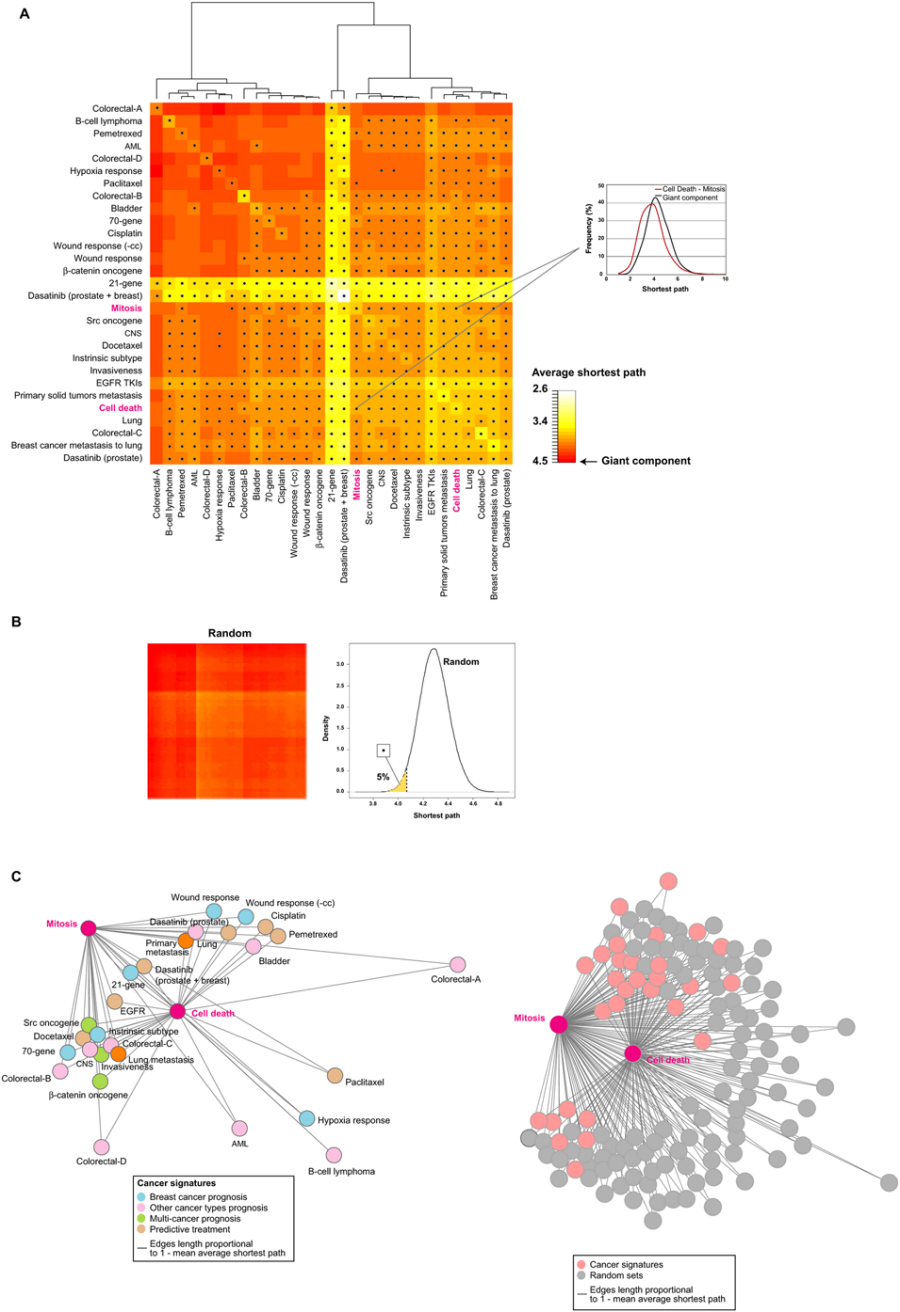
Proximity between gene products of signatures in the interactome network. *A*, heat map of average shortest paths between proteins encoded by signatures. This analysis was performed using only the giant network component. An example of shortest path differences with respect to the giant component is shown in the right panel for the comparison between the complete Cell Death and Mitosis GO sets. *B*, heat map of comparisons of 1,000 randomly selected 50-protein sets in the giant component. Right panel, density plot of average shortest path in randomly selected sets: the 5% lower values are highlighted, which correspond to an average shortest path <4.09. Comparisons between signatures below this empirical cut-off are shown by dots in *A*. *C*, left panel, network representation of average shortest paths between Cell Death and Mitosis and cancer signatures as shown in the inset. Edges lengths are proportional to the average shortest path values. Right panel, network representation of average shortest paths between Cell Death and Mitosis and cancer signatures or randomly selected protein sets with equivalent degree centrality.

All signature comparisons showed shortest path distributions skewed toward smaller values than expected from the giant component (Fig. 5). Statistical evaluation using the non-parametric MW test identified significant differences with respect to the giant component distribution in 90% of comparisons. The smallest shortest paths were identified for the 21-gene prognostic, and dasatinib and EGFR TKI predictive signatures, although the results may be subject to bias because these sets contain several proteins that are widely studied in the literature and therefore have high network centrality.

To further evaluate these differences, we randomly selected 1,000 sets of 50 proteins with similar average degree centrality to the signatures and obtained their shortest path distributions. Most of the cancer signatures were more closely located than expected by chance and also close to the Cell Death and Mitosis complete sets (empirical *P* values<0.05 marked with dots in Fig. 5*A and B*). According to these observations, examination of GO annotations in the direct and one-hop neighborhoods of signatures identified significant over-representation of Cell Cycle or Cell Death terms or their children in all cases (FDR-adjusted *P* values<0.05) (GO term details not shown), which reinforces the hypothesis that the signature gene products are molecularly and functionally associated with these processes.

Next, signatures were depicted as nodes in a network in which the length of the edges is proportional to the average shortest path to the Cell Death and Mitosis sets (Figure 5*C*, *left*). In this network, most signatures were found close to these central processes when compared to 100 random sets with equivalent degree centrality (Figure 5*C*, *right*). Distant signatures represented modest associations at the different molecular levels examined above, such as the prognostic signatures for B-cell lymphoma, colorectal cancer and hypoxia response. These observations suggested correlation across different molecular levels. Thus, negative correlations for all signatures were observed between PCC co-expression values and interactome shortest path distances (average *r* = −0.31 and *σ* = 0.16; Mantel test *P* value = 0.059), which is consistent with functional relationships [42]–[45]. Consequently, higher co-expression between signatures partially correlated with smaller shortest paths between them in the interactome network. These observations highlight the importance of the integrative study, which revealed previously unidentified relationships in gene lists.

The immune response signature was also located close to the Cell Death and Mitosis sets (MW test *P* values<0.001) (Figure S1*A*). Consequently, examination of the proportion of GO annotations in the one-hop neighborhood of this signature identified over-representations of terms related to cell proliferation and death, while the direct interactors only showed over-representation of terms associated with the immune system (Figure S1*B*). Thus, although the gene products with prognostic value for ER-negative breast cancer are not directly connected to the common processes identified above, they are significantly associated in a second term, as well as transcriptionally co-expressed and co-regulated with many signatures.

### Evaluation of properties and interactions in independent datasets

The observations described above were evaluated in two independently generated signatures of cancer conditions. A recent study described a lung metastasis signature of breast cancer using a different methodological approach [46]. We found ∼70% (15/21) of the genes in this signature to contain E2F TRANSFAC motifs and ∼60% (13/21) to be targets of E2F1-AP2 and/or ER. In addition, significant correlations with eight prognostic signatures were identified, seven of them of breast cancer (empirical *P* values<0.001) (results of the analyses of this signature are detailed in Table S2). The correlation with Mitosis was higher than expected (empirical *P*<0.001), while the correlation with Cell Death was non-significant (empirical *P* = 0.18). Finally, gene products in this signature showed smaller average shortest paths than expected with 21 of the 28 signatures, including Cell Death, Mitosis and the lung metastasis signature presented previously [31] (empirical *P* values<0.05).

**Figure 6:**
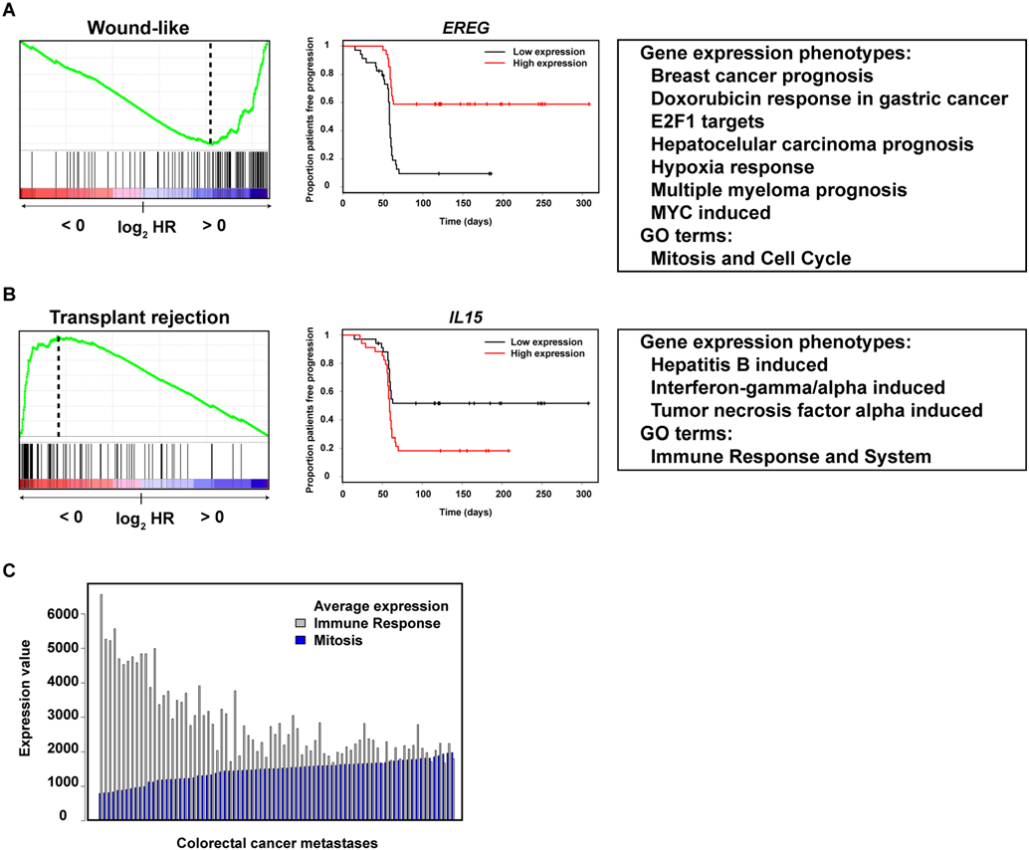
Asymmetric distribution of gene annotations in the response to cetuximab treatment. *A*, left panel, GSEA results for the strongest associated phenotype with high-expression genes predicting treatment response (log_2_ HR>0). Central panel, expression analysis plot of the extreme gene expression (*EREG*), which was also noted in the original publication [47]. Right panel, additional phenotypic and GO term sets with high-expression genes associated to treatment response at FDR *Q* values<1%. *B*, left panel, GSEA results for the strongest associated phenotype with low-expression genes predicting treatment response (log_2_ HR<0). Central panel, expression analysis plot of the extreme gene expression (*IL15*). Right panel, additional phenotypic and GO term sets with low-expression genes associated to treatment response at FDR *Q* values<1%. *C*, Histogram plot of average expression values of genes annotated with the Immune Response or Mitosis across samples in the cetuximab dataset. Average GO set expression values show a negative correlation with ordered metastatic samples.

To further corroborate our observations, we selected a different neoplastic condition from the recent literature: metastatic colorectal cancer treated with the EGFR inhibitor cetuximab (Erbitux®) [47]. Previous studies suggest that EGFR mutations are associated with the response to TKIs but not to cetuximab [48], [49]. We evaluated our observations by examining the distribution of gene annotations in the rank of hazard ratios (HRs) that measures the response to cetuximab treatment by progression-free survival. In this analysis, cell proliferation and the immune response were identified as the processes with the greatest effect on the response (Fig. 6). Importantly, the set of genes whose high expression most strongly associate to response was for a wound-like phenotype that was previously shown to provide prognosis value for breast, lung and gastric cancer [50]. The next associated high-expression sets were for doxorubicin treatment in gastric cancer, breast cancer prognosis (70-gene) and prognosis of different cancer types not examined in this study (hepatocellular carcinoma and multiple myeloma prognosis). Moreover, high-expression of E2F1, hypoxia and MYC targets was also associated with the response with similar strength (Fig. 6*A*). Collectively, these observations endorse the biological convergence of signatures.

The analysis of the cetuximab dataset also revealed a complementary behavior of cell proliferation and the immune response consistent with the representation of E2F1 motifs shown above. Patients with high expression of cell proliferation-related genes and low expression of immune response-related genes responded to treatment (Fig. 6*B*), whilst there were no patients with high expression values of both processes. Hence, a strong anti-correlation was observed between genes annotated with the GO term Immune Response and genes annotated with Mitosis (*r* = −0.79) (Fig. 6*C*). This observation leads to speculate that these processes play balancing roles in prognosis and treatment response. Good responders to cetuximab may show strong dependence on a “cell proliferation-on” molecular program, while non-responders could be sensitive to immune system-based therapy.

## Discussion

Despite the low degree of overlap in terms of gene identity, apparently dissimilar cancer signatures converge on specific biological processes. Convergence is defined by significant molecular and functional associations between genes and/or proteins: i/ predicted promoter motifs; ii/ experimentally identified DNA binding sites; iii/ cell cycle-periodic profiles; iv/ ER-mediated transcriptional regulation; v/ co-expression with defined transcription factors; vi/ co-expression between signatures and with specific GO gene sets; and, vii/ close proximity in the interactome network and neighborhood over-representation in these same GO terms. Consequently, this study suggests the existence of common design principles in a system-level cellular model—illustrated by transcriptome-interactome correlations—not only of prognostic signatures but also of metastasis and treatment response signatures. Overall, the integrative study highlights the importance of analyzing signatures beyond gene names, which provides a better global understanding by revealing previously unidentified properties and associations.

Biological convergence has important implications for the interpretation of signatures. Given a single gene whose transcript levels are associated with differences in patient outcome, this observation should be interpreted *a priori* in the context of cell proliferation, death or the immune response processes. For example, *BRCA1* and *BRCA2* have different cellular functions, with a degree of overlap, but each of them is present in several prognostic and predictive signatures, probably because their transcript levels reproduce precisely the potential for cell proliferation. This potential is defined by the presence of genes with periodic expression through the cell cycle, and other analyses at the genome, transcriptome and proteome levels shown here provide strong evidence of common properties and interactions. Therefore, further conclusions concerning gene functions such as DNA repair and its role in prognosis should be considered, controlling for the possible confounding effect of biological convergence.

From a mechanistic point of view, this study indicates the existence of a cancer cell phenotype that decisively influences critical aspects of neoplasia. This observation follows on from the long-known global importance of the potential for cell proliferation and repressed cell death in tumorigenesis [51], while reinforcing the emerging role of the immune response in prognosis and prediction [39]. However, while this study provides the first evidence of convergence of prognostic, metastasis and predictive signatures in these processes, other processes or signaling pathways are probably represented and specificities may exist. For instance, the potential for metastasis also depends on the activity of processes such as extracellular matrix remodeling. Similar systems-level analyses of a larger number of metastasis signatures may reveal properties masked here by the restriction of the study to mainly prognostic and predictive sets. Nonetheless, some prognostic or predictive sets are not independent of the potential for developing metastasis [10]. Future research may reveal a more complex molecular wiring diagram of the processes participating in cancer signatures.

## Materials and Methods

### Cancer signatures

We compiled 28 signatures from 24 studies, comprising 19 prognostic signatures, two signatures focused mainly on metastasis, and seven predictive treatment response signatures, as detailed in Table S1. Note that the 21-gene breast cancer prognosis signature was originally described as a predictive set for tamoxifen treatment [52] and the intrinsic subtype signature [53] corresponds to a validated set taken from the original report [13]. We also examined the wound response prognosis signature without including the initially identified cell cycle-associated genes [40] and the predictive signature for dasatinib treatment response subdivided for prostate and breast cancer [54]. Gene names or microarray probes were taken from the original publications and mapped to Entrez GeneIDs using the BioMart and Bioconductor [55] tools and by manual curation of each signature.

### Genomic analyses

Transcription factor (TF) motifs in promoter sequences 1 kilobase (kb) upstream of the transcription start site were predicted using MatScan [56] and position weight matrices from JASPAR [57] and TRANSFAC [58] (111 and 625 motifs, respectively). Probabilities were calculated using the Poisson distribution as an approximation to the binomial as follows 
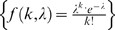
 (where 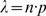, 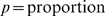 of genes with a defined motif that are part of the signature and *n* = total number of genes with this motif in the genome). Promoter sequences (−1 kb) of Ensembl protein-encoding gene entries (*n*≈18,800) were used as a common reference for the motif analyses. Corrections for multiple comparisons were computed using the false discovery rate (FDR) approach [59]. Chromatin immunoprecipitation data and periodically expressed genes were taken from the respective references [23], [24], [28] or from the relevant repositories [29] and examined using the same methodology. The ER binding sites identified by chromatin immunoprecipitation assays were assigned to a single GeneID based on the closest known gene locus (5′-end) in the May 2004 version of the human genome in the UCSC Genome Browser.

### Transcriptomic analyses

Transcriptional targets of the ER signaling pathway were examined using preprocessed and normalized data [29]. Correlations of transcription factors were performed by defining differentially expressed genes at FDR<1% in breast cancer prognosis measured with metastasis events within 5 years [12], which correspond to 179 microarray probes, or by defining differentially expressed genes at FDR<5% in docetaxel treatment response [37], which correspond to 1,525 probes. Differences in PCC distributions were assessed using the Mann-Whitney (MW) and Kolmogorov Smirnov non-parametric tests, with similar results. Average correlations in pairwise signature comparisons were calculated using all probes in the signature gene lists and compared to equivalent probe sets randomly selected from the same breast cancer dataset [40]. Dimensionality was reduced by applying a principal component analysis (PCA) until ∼80% of the variance in gene expression was captured, which represented <25 genes in the Cell Death or Mitosis Gene Ontology (GO) sets (originally containing 58 and 117 genes, respectively). For the analysis of cetuximab treatment response, we computed a Cox proportional hazards model for each microarray probe, using the progression-free survival as the time variable, and dividing the sample set into two equally-sized groups according to the expression level of the corresponding probe (low versus high). Ranks according to the log-hazard ratio were used as input lists for the Gene Set Enrichment Analysis (GSEA) [60]. The GSEA was run for all curated and GO datasets found in MSigDB database. We used default values for all the parameters except for the median probe instead of the max probe as the collapse method when multiple probe sets map to the same gene. The evaluation of correlation between the Immune Response (*n* = 311) and Mitosis GO sets in the dataset of cetuximab treatment response was performed averaging expression values of both gene sets in each metastasis sample. The R programming language was used for analyses and graphics.

### Interactome analyses

The human interactome network was built by combining three previously published datasets consisting mainly of experimentally verified interactions [41]. The dataset based on the Human Protein Reference Database (HPRD) contains compiled and filtered binary protein interactions from available databases. High-confidence yeast two-hybrid interactions were then incorporated and orthology-based predictions and homodimers were excluded to avoid specific bias. Proteins with no assigned GeneID were also excluded from our analyses. The numbers of proteins or nodes and interactions or edges in the complete dataset were 8,519 and 35,492, respectively. The percentage of signature gene products mapped in this dataset ranged between 40 and 85. Shortest paths were calculated using only the giant network component and the geodesic formulation given by Freeman in the R programming language [41]. Differences in the distributions of shortest paths were assessed using the MW test. Empirical simulations using 50-protein sets were selected as the average size of cancer signatures, using only nodes from the giant component with average degree centrality equivalent to the signatures. The average degree of signatures, excluding three outliers that contain widely studied genes (21-gene, dasatinib prostate and breast, and EGFR TKIs), was 7.48, while the average degree of 1,000 random sets was 7.53. To evaluate the relationship between gene co-expression and interactome distances, a correlation coefficient was calculated between average PCCs in each signature-pair and the corresponding average short path in the giant network component, which was then evaluated to the null hypothesis of no-correlation between the two measures using the Mantel test. The representation of GO terms in neighborhoods was assessed using the shortest path measure and the hypergeometric distribution and FDR *P* value adjustment, taking as a reference all proteins in the giant component and excluding signature proteins in each case. The Onto-Express tool was used for this analysis [61].

## Supporting Information

Figure S1 Topological associations of the immune response signature in the interactome network. A, left panel, shortest path distributions between the immune response and the Cell Death and Mitosis sets (yellow and green curves, respectively) relative to the giant component (black curve). Right panel, strategy for evaluating differences in proportions of GO annotations in the direct and one-hop interactome network neighborhoods. B, over-represented GO terms in the direct and one-hop neighborhoods of the immune response signature.
(2.22 MB EPS)

Table S1 (0.03 MB XLS)

Table S2 (0.03 MB XLS)
